# E-vita Open Neo Hybrid Stent Graft Implantation Technique

**DOI:** 10.1177/15569845251364258

**Published:** 2025-08-21

**Authors:** Ryaan EL-Andari, Michael C. Moon

**Affiliations:** 1 Division of Cardiac Surgery, Department of Surgery, University of Alberta, Edmonton, AB, Canada

Management of the aortic arch has evolved greatly over the preceding decades. The frozen elephant trunk (FET) procedure has evolved into hybrid devices that allow for replacement of the arch and descending thoracic aorta.^1–3^ The E-vita Open Neo (Artivion, Kennesaw, GA, USA) is an updated version of the original E-vita device that has been gaining popularity. In this video report, we describe the key features of implantation of the E-vita Open Neo.

A 44-year-old male patient with a medical history of acute type A dissection repair with a hemiarch replacement and AMDS Hybrid Prosthesis (Artivion) implantation 3 years prior presented with an expanding aortic arch aneurysm measuring 61 mm. He was put forward for a total arch replacement with FET. The plan included a zone 0 FET and reimplantation of the head vessels to avoid excising the arch stent.

The case setup included a left femoral artery puncture with delivery of a pigtail catheter into the true lumen with location confirmed by transesophageal echocardiogram used to guide the FET into the true lumen, left axillary cannulation with an 8 mm graft, redo sternotomy, systemic hypothermia with cooling to 25°C, hypothermic circulatory arrest, and cannulation of the innominate artery with a 3.5 mm Stockert cannula.

Once the setup was complete, the previous aortic graft was incised, and the wire placed via the left femoral artery was retrieved. The device was then delivered over the wire and placed in situ. The wire was removed, and the device was deployed. The distal anastomosis was completed using a running 4-0 Prolene suture. A custom-made trifurcated Dacron graft was created for reconstruction of the head vessels. The graft was brought into the field, and the innominate anastomosis was created using the 12 mm limb with a running 4-0 Prolene suture. The previous graft-to-graft anastomosis was taken down, and the proximal anastomosis of the E-vita graft was placed just below the sinotubular junction using a 4-0 Prolene suture. A side-biting clamp was applied to the anterolateral aspect of the main body of the aortic graft, and using a hot tip cautery, an aortotomy was performed in the Dacron graft to which we constructed the 12 mm segment of the trifurcated head vessel branch graft using a 5-0 Prolene suture. The 8 mm side limb from the left axillary artery was then divided and brought into the left pleural space and into the mediastinum. The graft was then anastomosed in an end-to-end fashion to the trifurcated, custom-made graft using a 5-0 Prolene suture. The patient was then weaned from cardiopulmonary bypass, hemostasis was confirmed, and the chest was closed. The patient was returned to the cardiovascular intensive care unit.

This video report demonstrates the surgical technique for the implantation of the E-vita Open Neo hybrid aortic arch FET in a complex case with a previous aortic dissection repair with AMDS Hybrid Prosthesis implantation (Supplemental Video).
